# Non-Invasive Electrochemical Biosensors for Fibromyalgia: A Path Toward Objective Physiological Monitoring and Personalized Management

**DOI:** 10.3390/s26082301

**Published:** 2026-04-08

**Authors:** María Moreno-Guzmán, Juan Pablo Hervás-Pérez, Edurne Úbeda-DÒcasar, Marta Sánchez-Paniagua

**Affiliations:** 1 Department of Chemistry in Pharmaceutical Sciences, Faculty of Pharmacy, Complutense University of Madrid, 28040 Madrid, Spain; marimore@ucm.es (M.M.-G.); jphervas@ucm.es (J.P.H.-P.); 2 FIBYSAR Research Group, Faculty of Health Sciences, HM Hospitals, University Camilo José Cela, Urb. Villafranca del Castillo, 49, Villanueva de la Cañada, 28692 Madrid, Spain; eubeda@ucjc.edu; 3 Instituto de Investigación Sanitaria, HM Hospitales, 28015 Madrid, Spain

**Keywords:** fibromyalgia, electrochemical biosensors, DNA probes, salivary biomarkers, central sensitization, personalized medicine, point-of-care diagnostics

## Abstract

Fibromyalgia (FM) is a complex chronic syndrome marked by widespread musculoskeletal pain, neurocognitive dysfunction (“fibro-fog”), and autonomic disturbances. Clinical management remains challenging due to subjective symptom reporting and the lack of definitive diagnostics. Emerging evidence points to a multifactorial origin involving central sensitization, neuroendocrine imbalance, and systemic immune-inflammatory alterations. A wide array of candidate biomarkers has been reported in FM, encompassing neurotransmitters (serotonin, norepinephrine), excitatory and inhibitory amino acids, metabolic and glycolytic enzymes, stress-related proteins, autoantibodies, oxidative stress markers and pro-inflammatory cytokines. This molecular heterogeneity reflects the systemic and multidimensional nature of FM. However, most of these biomarkers have been primarily investigated in serum or plasma, where analytical validation and reference ranges are more established. In contrast, the exploration of salivary biomarkers—although highly attractive due to its non-invasive, stress-free, and repeatable collection—remains comparatively limited. Saliva contains a reduced concentration range of many systemic markers and is strongly influenced by circadian rhythms, stress, flow rate, and oral health conditions. While promising candidates such as α-amylase, cortisol, calgranulins, and selected metabolic enzymes have shown potential in saliva, many proposed FM-related biomarkers lack full analytical validation, standardized protocols, and clinically defined reference intervals in this matrix. In this context, non-invasive electrochemical biosensors represent a transformative technological approach. Advanced electrode architectures incorporating nucleic acid probes, redox reporters, and nanostructured materials offer high sensitivity in low-volume and low-concentration biofluids such as saliva. The integration of multiplexed biomarker panels into portable platforms could enable real-time, longitudinal monitoring of FM pathophysiology, supporting phenotype stratification, personalized therapeutic adjustment, and objective disease activity tracking.

## 1. Fibromyalgia: Background and Pathophysiology

Fibromyalgia (FM) is a multifaceted, chronic syndrome principally defined by widespread musculoskeletal pain, but it also encompasses persistent fatigue, sleep disturbances, cognitive impairment (“fibro-fog”), tenderness, and heightened multisensory sensitivity. While FM was historically conceptualized as a disorder of abnormal pain processing, contemporary research has elucidated a far more intricate pathophysiology. FM disproportionately affects women, with a prevalence that is substantially higher in females than in males, raising important considerations regarding the influence of sex-specific biological and sociodemographic factors on disease expression. For example, in a large prospective cohort of Norwegian women, Benebo et al. [1] reported that overweight and obesity, very low physical activity, smoking, and patterns of alcohol consumption were each associated with a significantly increased risk of self-reported FM, emphasising the contribution of lifestyle and body composition to disease risk in women. These findings highlight that sample characteristics beyond mere diagnosis—such as BMI, physical activity levels, and other lifestyle determinants—may confound phenotypic expression and should be carefully characterised in FM research. Moreover, emerging evidence suggests that the menopausal transition may modify the clinical phenotype of FM, with symptom severity and impact often fluctuating in relation to hormonal status; Clarke et al. [2] discuss how the menopause transition may influence pain and functional outcomes, underscoring the need to consider reproductive lifespan and hormonal milieu when interpreting results in predominantly female cohorts. Additionally, recent work on stress physiology in FM indicates that perceived stress is elevated in affected individuals, although biomarker measures such as salivary or hair cortisol do not consistently differ from controls, reflecting the complex and often discordant relationship between subjective stress experience and HPA (Hypothalamic–Pituitary–Adrenal) axis biomarkers in FM [3]. Therefore, detailed characterisation of sociodemographic variables, lifestyle behaviours, hormonal status, and neuroendocrine profiles is essential for improving the validity and generalisability of research findings, and for disentangling the multifactorial underpinnings of FM [4].

Central sensitization (characterized by an amplified responsiveness of the central nervous system to nociceptive stimuli) remains a central paradigm, explaining the augmented pain perception in the absence of clear peripheral tissue damage [5,6,7,8,9,10]. However, the exclusive focus on neuronal mechanisms is insufficient to account for the full clinical spectrum of FM, which includes profound fatigue, neurocognitive dysfunction, and autonomic symptoms. Recent integrative models propose that FM arises from the interplay of central sensitization, neuroendocrine dysregulation, immune-inflammatory alterations, oxidative stress, metabolic disturbances, and neuroimmune interactions [10,11,12,13]. At the molecular level, FM is associated with elevated proinflammatory cytokines (e.g., IL-6, TNF-α), altered levels of neuropeptides such as substance P (SP), dysregulation of neurotransmitters including serotonin (5-HT), dopamine (DA), and norepinephrine (NE), and markers of oxidative stress. Genetic predispositions and epigenetic modifications may influence susceptibility and symptom severity. Mitochondrial dysfunction and impaired energy metabolism are proposed mechanisms underlying persistent fatigue and cognitive deficits. This holistic perspective recognizes FM as a multisystem disorder, likely comprising heterogeneous subtypes—such as those with predominant central sensitization features, immune/inflammatory features, metabolic or neuroendocrine involvement—which may overlap in individual patients [14]. Recognition of these molecular and metabolic pathways may inform the identification of specific biomarker panels for disease monitoring and the development of targeted therapies for distinct FM subtypes.

Given this complexity, there is a pressing need for objective biomarkers to monitor disease activity, symptom fluctuations, and therapeutic response, moving beyond reliance on subjective symptom reporting [11,12,13,14].

However, systemic biomarkers alone may be insufficient to capture the central mechanisms underlying FM. To fully comprehend the biological heterogeneity of FM, it is essential to integrate peripheral measures with central and environmental factors. Neuroimaging techniques, such as functional (fMRI) and positron emission tomography (PET), have identified consistent alterations in brain networks involved in pain processing and modulation, as well as in neurotransmission systems related to central sensitization, positioning these approaches as relevant complementary biomarkers in FM [15,16]. In this context, proton magnetic resonance spectroscopy (^1^H-MRS), widely used in neurobiological disorders such as depression, schizophrenia, or autism, has recently been applied to FM research, with a limited number of studies to date. ^1^H-MRS allows in vivo assessment of both static and dynamic levels of key neurotransmitters, including glutamate and γ-aminobutyric acid (GABA), providing insight into excitatory-inhibitory balance in brain regions involved in pain generation and amplification. The gut indicates an increased excitatory/inhibitory ratio in areas such as the somatosensory cortex, insula, and anterior cingulate cortex, contributing to the hypersensitivity characteristic of FM.

Furthermore, combining ^1^H-MRS with pharmacological interventions offers high translational potential by allowing direct mapping between underlying pathophysiology and drug mechanisms, potentially facilitating the discovery of novel therapeutic compounds and improving personalized chronic pain management in FM patients [17,18,19].

Beyond neuroimaging, epigenetic mechanisms may contribute to the biological heterogeneity of FM. Epigenetics, including DNA methylation, histone modifications, and microRNA expression, regulates gene activity without altering the DNA sequence, linking environmental exposures to gene expression and health outcomes [20,21]. Epigenetic changes can modify chromatin structure and increase the frequency of somatic chromosomal abnormalities, as observed in stress-related conditions. The cytokinesis block micronucleus (CBMN) assay is a reliable method to quantify these alterations in somatic cells. In a pilot study, the frequency of spontaneously occurring micronuclei (MN) and genome-wide methylation patterns were compared in women with FM (n = 10) versus age-matched healthy controls (n = 42 [MN]; n = 8 [methylation]). Women with FM showed a significantly higher MN frequency (mean (SD) 51.4 (21.9)) than controls (15.8 (8.5)) (χ^2^ = 45.552; df = 1; *p* = 1.49 × 10^−11^) [22]. Significant differences were observed in 69 methylation sites, with 91% showing increased values in FM patients. These sites were enriched in biological pathways related to neuronal differentiation, nervous system development, skeletal and organ system development, and chromatin compaction. Genes of relevance included BDNF, NAT15, HDAC4, PRKCA, RTN1, and PRKG1 [23,24]. These findings support the hypothesis that acquired epigenetic and chromosomal alterations may underlie the biological mechanisms of FM.

Additionally, gut microbiota has been proposed to influence FM pathophysiology. The microbiome is essential for host health through metabolic, immunological, and gut-protective functions, including the production of short-chain fatty acids (SCFAs), which maintain intestinal barrier integrity and support blood–brain barrier development. Dysbiosis, defined as differences in microbial composition between healthy individuals and disease-specific patients, has been linked to FM symptoms, including pain, fatigue, and mood disturbances. The gut–brain axis, a bidirectional communication system involving neural and humoral pathways, provides a mechanism connecting microbial activity with central nervous system function. For example, microbial GABA production from glutamate may influence host behavior, contributing to anxiety and depression. Altered microbiota can also enhance immune activation via pro-inflammatory cytokines, some of which increase blood–brain barrier permeability, potentially promoting central sensitization in FM. Integrative omics approaches combining microbiome, genome, epigenome, transcriptome, and metabolome data show that host genetics can shape microbiome composition, while microbiome-derived metabolites can modulate systemic and neural physiology [25,26,27,28]. These findings suggest that microbiota alterations may interact with genetic and epigenetic factors, contributing to the heterogeneity and severity of FM symptoms, and highlight the potential of targeting the gut microbiome in future therapeutic strategies.

While epigenetic regulation and gut microbiota alterations are increasingly recognized as contributors to FM pathophysiology, their relevance for biosensing applications depends on the identification of measurable downstream analytes. Epigenetic mechanisms may influence the expression of circulating microRNAs, inflammatory mediators, or stress-related hormones that can be detected in accessible biofluids such as saliva or sweat. Similarly, microbiota dysbiosis has been associated with altered production of metabolites and immune signaling molecules that may also appear in peripheral biofluids. Several of these molecular species—including cytokines, neurotransmitter-related metabolites, and small regulatory RNAs—are compatible with electrochemical detection strategies using antibodies, aptamers, or nucleic acid probes. Therefore, although epigenetic and microbiome alterations are complex systemic processes, they may still contribute to the development of non-invasive biosensing targets through their measurable biochemical outputs.

## 2. Biomarkers Relevant for Monitoring Fibromyalgia

Despite substantial advances in understanding FM, the identification of robust and objective biomarkers suitable for disease monitoring remains a major challenge. FM is a heterogeneous and multifactorial syndrome, and its clinical complexity is reflected in the diversity of molecular, neurochemical, immune, and metabolic pathways implicated in its pathophysiology; consequently, no single biomarker is sufficient to capture the full biological spectrum of the disorder. Current research therefore supports a multidimensional biomarker framework encompassing neurotransmitters (acetylcholine, blood NE, DA, epinephrine, 5-HT, SP), amino acids (glycine, glutamate, serine), enzymes involved in oxidative stress and energy metabolism (α-amylase, α-enolase, PGAM1, TALDO1), pro- and anti-inflammatory cytokines (IL6, IL8, IL10, TNFα, CCL2, CCL5, CXCL9, CXCL10), non-enzymatic proteins (IgG anti-satellite glial cell antibodies, calgranulin A/C, serotransferrin), and other relevant biomolecules (ascorbic acid, uric acid, cortisol), assessed in blood, saliva, or cerebrospinal fluid. Organizing these candidate biomarkers according to their primary biological function—neurochemical, immune, metabolic, or oxidative—facilitates mechanistic interpretation, the integration of heterogeneous findings, and a more coherent assessment of their potential clinical relevance, while enabling longitudinal monitoring of disease activity, symptom progression, and therapeutic response. Table 1 summarizes these candidate biomarkers, their classification by biological function, and the sample types in which they can be measured.

Although numerous candidate biomarkers have been reported in FM, many findings remain inconsistent across studies due to limited cohort sizes, methodological heterogeneity, and significant inter-individual variability. Consequently, only a subset of these markers currently demonstrates sufficient reproducibility and biological plausibility to be considered realistic targets for biosensor-based clinical monitoring.

From a biosensing perspective, the suitability of these biomarkers for electrochemical detection depends not only on their pathophysiological relevance but also on their stability in accessible biofluids, their typical concentration ranges, and the availability of selective recognition strategies such as antibodies, aptamers, or enzyme-based detection schemes. Consequently, biomarkers present in saliva or sweat at concentrations compatible with the detection limits of current electrochemical platforms represent particularly promising candidates for non-invasive monitoring in FM.

### 2.1. Neurotransmitters and Neuromodulators

Alterations in neurotransmitter systems, particularly serotonin and dopamine, have been proposed to contribute to the pathophysiology of FM and may influence pain modulation and central sensitization. Alterations in neurotransmitter systems are a central component of FM pathophysiology, providing a biological substrate for its core clinical manifestations, such as widespread pain, fatigue, sleep disturbances, cognitive dysfunction, and affective symptoms. These abnormalities reflect a complex imbalance between excitatory and inhibitory neuromodulatory systems. Among these, catecholaminergic and indolaminergic pathways have received particular attention; in controlled studies, FM patients exhibit significantly higher plasma NE levels alongside reduced concentrations of 5-HT, DA, and their metabolites compared with healthy controls, suggesting a global dysfunction that contributes to clinical symptomatology.

FM is a complex syndrome characterized by widespread pain, unclear etiology, and difficult diagnosis. Among the most extensively studied biological alterations is the dysregulation of the HPA axis and the sympathetic nervous system (SNS) [29]. Although meta-analyses report inconsistent findings for cortisol, ACTH, and CRH, elevated plasma NE is a relatively frequent finding, suggesting increased sympathetic tone associated with relative adrenocortical hypofunction [11,30].

5-HT is the most widely studied neurotransmitter in FM. Reduced peripheral and central serotonergic activity has been consistently associated with enhanced pain perception, mood disturbances, and sleep disorders. These observations support the clinical efficacy of serotonergic agents and dual 5-HT–NE reuptake inhibitors. In parallel, DA plays a key role in endogenous pain modulation and reward-related circuits, and available evidence points to dopaminergic hypofunction associated with fatigue, anhedonia, and cognitive impairment [31].

In this context, the study of monoaminergic neurotransmitters has provided relevant insights. Analyses of catecholamines (epinephrine, NE, and DA), as well as indolamines and intermediary metabolites of 5-HT, have revealed significant alterations in women with FM. Compared with healthy controls, higher plasma NE levels and lower concentrations of DA, 5-HT, 5-HIAA, and 5-HTP have been reported, with no significant differences in epinephrine or N-acetyl-5-HT. Moreover, plasma NE has been associated with poorer physical status, and concentrations above 694.69 pg/mL have been proposed as a potential predictor of FM, reinforcing the link between sympathetic hyperactivity and clinical severity [31].

Taken together, these findings support the existence of dysregulation in catecholaminergic and indolaminergic pathways in FM, with clear pathophysiological relevance and potential diagnostic utility [11,29].

Neuropeptides and amino acids further characterize the “excitatory bias” of FM. SP, a key mediator of nociceptive transmission, is consistently reported at elevated levels in the CSF of FM patients, supporting its role in central sensitization [32]. This is complemented by elevated concentrations of excitatory amino acids, such as glutamate and glycine, in brain regions involved in pain processing. Importantly, accumulating evidence indicates that this excitatory bias is exacerbated by reduced inhibitory GABAergic neurotransmission. Neuroimaging studies using magnetic resonance spectroscopy have reported decreased GABA levels in key pain-processing regions, including the insula and anterior cingulate cortex, linking deficient descending pain control with increased pain sensitivity.

Finally, acetylcholine (ACh) plays a key role in the cholinergic anti-inflammatory pathway (CAP) and in cognitive function, and its dysregulation may contribute to both impaired attentional control (“fibro fog”) and inadequate modulation of inflammatory responses in fibromyalgia syndrome (FMS). However, its use as a clinical biomarker is limited by rapid enzymatic degradation and the difficulty of accessing representative central compartments, such as cerebrospinal fluid, so direct measurement is largely restricted to experimental studies. For this reason, choline, a stable precursor of acetylcholine, is used as an indirect marker of cholinergic function. Serum choline levels have been reported to be lower in patients with FMS, particularly in newly diagnosed drug-naive individuals, and choline concentrations show a weak-to-moderate positive correlation with dietary intake, which is also generally lower in these patients compared to healthy controls [33]. These findings support the hypothesis that cholinergic dysfunction, reflected indirectly through choline and functional or molecular markers, may contribute to the pathophysiology of FMS and highlight the importance of integrating these measures into multimodal biomarker panels to identify biologically meaningful subtypes of the disorder.

### 2.2. Amino Acids

Beyond their role in neurotransmission, amino acids represent key metabolic indicators that are significantly dysregulated in FM. Multiple metabolomic studies have demonstrated alterations in serum amino acid profiles in FM patients compared with healthy controls.

Several amino acids involved in energy metabolism and nitrogen balance, including alanine (Ala), arginine (Arg), citrulline (Cit), glutamine (Gln), proline (Pro), serine (Ser), α-aminopimelic acid (α-APA), and hydroxyproline (Hyp) have been reported at lower levels in FM patients. In contrast, increases have been described for other amino acids and related metabolites, such as aromatic amino acids (AAA), asparagine (Asn), threonine (Thr), methionine (Met), ornithine (Orn), lysine (Lys), and methylhistidine derivatives (3-MH and 5-MH) [34]. These findings suggest that amino acid metabolism may play a relevant role in the pathophysiology of FM and may provide potential biomarkers for prognosis, treatment, and disease monitoring.

The observed amino acid imbalances likely reflect a state of altered energy metabolism, mitochondrial dysfunction, and impaired neurotransmitter synthesis. Amino acids such as glutamate and glycine act as a bridge between neurochemical and metabolic pathways, functioning both as metabolic substrates and central signalling molecules. This dual role emphasizes the interconnected nature of FM pathophysiology, where metabolic disturbances may contribute to central sensitization and cognitive dysfunction [34,35,36,37,38].

Furthermore, amino acid alterations are highly sensitive to biological variables; profiles may differ according to sex, menopausal status, BMI, and specific clinical phenotypes. This variability highlights the potential utility of amino acid profiling for patient stratification and personalized monitoring rather than universal diagnosis. The identification of these metabolic fingerprints may facilitate the development of targeted interventions and objective measures of disease progression.

### 2.3. Enzymes

Proteomic analyses have identified several enzymes involved in glycolysis, redox homeostasis, and physiological stress responses as differentially expressed in FM, particularly in saliva. Among these, α-enolase and phosphoglycerate mutase 1 (PGAM-1) stand out as key glycolytic enzymes whose altered expression suggests impaired cellular energy metabolism and metabolic stress. Beyond its catalytic role, α-enolase possesses significant immunomodulatory properties; notably, autoantibodies against this enzyme have been reported in FM patients. This finding provides a compelling mechanistic link between metabolic dysfunction, immune activation, and central sensitization, all of which may collectively contribute to the maintenance of chronic pain [39,40,41].

Transaldolase 1 (TALDO1), a rate-limiting enzyme of the non-oxidative pentose phosphate pathway, has emerged as a particularly relevant biomarker in FM. Proteomic studies consistently report increased salivary expression of TALDO1 in FM patients, suggesting a metabolic shift toward pathways involved in antioxidant defense. Given its role in maintaining NADPH availability, TALDO1 upregulation likely reflects a compensatory response to chronic oxidative stress and impaired energy metabolism, both central features of FM pathophysiology. Consequently, TALDO1 serves as a bridge between metabolic dysfunction, low-grade inflammation, and central pain sensitization, reinforcing its value for disease monitoring and patient stratification [39,42,43,44].

In parallel, α-amylase—specifically when measured in saliva—is consistently elevated in FM [45,46], and is widely interpreted as a robust marker of salivary sympathoadrenal activity and the systemic stress response [44,47]. Due to its reproducibility and the feasibility of non-invasive assessment, salivary α-amylase represents one of the most promising biomarkers for real-time FM monitoring, reflecting the intricate integration of metabolic and autonomic dysregulation.

Together, these enzymatic alterations support the hypothesis that disrupted energy metabolism, oxidative stress, and heightened physiological stress responses drive the persistence of FM symptoms. Their greatest clinical utility resides in their inclusion within multimodal biomarker panels that integrate metabolic, neuroimmune, and autonomic data to enhance patient stratification and objectively monitor therapeutic response.

### 2.4. Cytokines and Chemokines

Low-grade immune activation has been described in specific subsets of FM patients, primarily reflected by altered cytokine profiles. In general, FM cohorts tend to show elevated levels of pro-inflammatory cytokines, particularly IL-6, IL-8, and TNF-α, together with occasional increases in the anti-inflammatory cytokine IL-10 e [41,48].

While the magnitude of these elevations may vary between studies, a consistent trend toward immune dysregulation has been reported. This variability likely reflects the inherent clinical heterogeneity of FM, the presence of comorbidities, and the influence of external modulators such as physical activity, sleep quality, and psychological stress. Notably, cytokine levels are highly dynamic and responsive to non-pharmacological interventions—particularly exercise—supporting their potential role as state-dependent biomarkers for longitudinal monitoring rather than stable diagnostic indicators [3,41,49].

Salivary cytokine profiling has emerged as a promising, minimally invasive approach to capture these fluctuations, although standardization challenges persist. Within this immune framework, chemokines constitute a pivotal subgroup involved in immune cell trafficking, neuroimmune communication, and the direct modulation of pain pathways. Increasing evidence suggests that chemokines link peripheral immune signaling with central sensitization mechanisms, oxidative stress, and metabolic dysfunction [50].

Among these mediators, CCL2 (MCP-1), CXCL9, and CXCL10 have received particular attention due to their roles in monocyte and T-cell recruitment, microglial activation, and neuroinflammatory signaling. These chemokines are inducible by pro-inflammatory cytokines such as IFN-γ and TNF-α, both of which are altered in FM cohorts. Dysregulated circulating levels of these molecules suggest a primed immune milieu that may contribute to sustained nociceptive signaling, heightened pain sensitivity, fatigue, and cognitive symptoms [51,52,53].

Emerging evidence indicates that several chemokines are detectable in non-invasive biological fluids, including saliva, reinforcing their utility as components of multimodal biomarker panels. Importantly, while group-level differences between FM patients and healthy controls are frequently described, chemokine alterations exhibit substantial interindividual variability. This heterogeneity supports the existence of immune-related FM subphenotypes, highlighting the relevance of chemokine profiling for patient stratification and the advancement of precision medicine approaches in chronic pain management.

### 2.5. Non-Enzymatic Proteins

Several non-enzymatic proteins have been implicated in FM, providing further insight into the complex immune, inflammatory, and metabolic dysregulation of the disorder. A growing body of evidence indicates that a subset of FM patients exhibits elevated IgG autoantibodies, including those targeting satellite glial cells and sensory neurons. These autoantibodies have been associated with increased pain sensitivity and central sensitization, supporting the emergence of an autoimmune-like mechanism in specific FM phenotypes [54].

Beyond immunological markers, serotransferrin—the principal iron-transport protein—has been reported at reduced levels in FM patients. This depletion suggests altered iron metabolism and enhanced oxidative stress, both of which may exacerbate fatigue, widespread pain, and neuroinflammatory signaling [39,41,55].

Furthermore, emerging evidence highlights the S100 family proteins as a novel class of biomarkers in FM. These small, low molecular weight proteins function as Damage-Associated Molecular Patterns (DAMPs), modulating diverse intra- and extracellular immune signaling pathways [56]. Among them, Calgranulin A/C (S100A8/S100A9) proteins are consistently overexpressed in FM saliva and are closely linked to inflammatory and oxidative stress pathways [42,44]. Their elevation reflects a critical interplay between immune activation, oxidative imbalance, and nociceptive sensitization, reinforcing their relevance as robust candidate biomarkers for non-invasive monitoring.

Together, these findings highlight those non-enzymatic proteins that act as integrative markers of the immune, metabolic, and oxidative shifts in FM. Their greatest clinical utility lies in their inclusion within multimodal biomarker panels, which combine proteomic, enzymatic, cytokine, and neurotransmitter data to improve patient stratification, monitor disease activity, and guide individualized therapeutic interventions.

### 2.6. Other Relevant Biomolecules

Certain biologically relevant molecules do not fit strictly within the previous categories but remain fundamental to understanding FM pathophysiology. Cortisol, the primary effector of the HPA axis, is among the most extensively studied biomarkers in FM, particularly in saliva. Altered diurnal rhythms—characterized by blunted cortisol awakening responses and reduced morning levels—are frequently reported. These fluctuations reflect dysregulated stress responses and autonomic imbalance, potentially contributing to the persistent fatigue and pain that define the disorder, although findings can vary depending on the clinical subtype [30,44].

Furthermore, markers of purine metabolism and antioxidant status provide insight into the systemic burden of the disease. Uric acid, a metabolic by-product with potent antioxidant properties, has shown variable associations with FM; however, elevated levels in certain chronic pain populations may reflect a compensatory response to heightened oxidative stress or altered metabolic flux [57]. Similarly, ascorbic acid (vitamin C), a critical endogenous antioxidant, plays a key role in maintaining redox homeostasis and modulating inflammatory pathways. While direct evidence linking circulating vitamin C levels to FM remains an area of ongoing research, its inclusion in multimodal panels is essential to capture the full scope of systemic oxidative stress and to complement the assessment of enzymatic and protein-based markers [39,44,57].

**Table 1:** A Comprehensive Compendium of Multi-Omic Biomarkers in Fibromyalgia.

	Biomarker	Analyzed Fluid	Biomarker Levels in FM Patients	Findings	Citations
**Neurotransmitters**	Acetylcholine (ACh)	Serum (blood)	Altered (direction unclear)	Indirect evidence of dysregulation	[58]
	Choline	Serum and Plasma	Decreased	There is no reference value due to the high variability among analytical methods.	[33]
	Norepinephrine (NE)	Plasma	Increased	Predictive threshold: 694.69 pg/mL. Dependent on stress, posture, and time of day.	[31]
	Dopamine (DA)	Plasma	Decreased	Altered dopaminergic activity. Highly variable; plasma measurements unstable.	[31]
		Urine			[59]
	Epinephrine (EN)/(Adrenaline)	Plasma	No significant change	Increases with stress or exercise	[31]
	Serotonin (5-HT)	Plasma	Decreased	No reference value due to dependence on sample processing and anticoagulant.	[31]
	Substance P (SP)	CSF	Increased	Levels were threefold higher in CSF than in controls, showing a weak correlation with tender point count.	[32]
**Amino Acids**	Amino Acids (total, profile)	Serum and plasma	Imbalances ↓ Ala|Arg|Cit|Gln|Pro|Ser|α-APA|Hyp ↑ AAA|Asn|3-MH|5-MH|Thr|Met|Orn|Lys)	↑/↓ (depends on amino acid) May reflect altered energy metabolism, neurotransmitter synthesis, and metabolic signaling.	[34,60]
**Enzyme**	α-amylase *	Saliva	Increased	Fluctuates with sampling time; may relate to pain and stress.	[45,46]
	PGAM-1 * (Phosphoglycerate mutase 1)	Saliva	Increased	Reported as significantly increased in patients with FM compared to healthy controls. Further validation studies are required	[42,44]
	Transaldolase 1 * (TALDO1)	Saliva	Increased	Consistently reported as elevated in FM patients. Potential role as a non-invasive biomarker	[39,42,43,44]
**Cytokines**	CCL2 (MCP-1)	Serum	Increased	Serum levels correlate positively with disease severity.	[51]
	CXCL9 (MIG)	Serum	Increased	Limited but suggestive evidence of elevation	[52]
	CXCL10 (IP-10)	Serum	Increased	Elevated in a subset of FM patients with C11 or F40 nonsense mutation	[53]
	IL-1 receptor antagonist (IL-1RA)	Serum	Increased	Possible dysregulation of cytokine signaling and an anti-inflammatory response in the disorder	[50]
	IL 1β/IL 1	Serum and skin biopsies	Increased	IL-1β levels may reflect an inflammatory component in pain induction in patients with FM	[61,62]
	IL-6	Serum, plasma and skin biopsies	Increased	Significantly associated with fatigue and pain ratings	[50,62]
	IL-8	Serum and CSF	Increased	Fatigue and depression were associated with high levels of IL-8, with the promotion of sympathetic pain	[50,63]
	IL-10	Serum	Increased	This increase also suggests the presence of an anti-inflammatory response and a link with clinical symptoms	[64]
	TNF-α	Serum and skin biopsies	Increased	Associated with an inflammatory component in the induction of pain	[62,65]
**Proteins (non-enzymatic)**	Antibodies (IgG, anti-satellite glial cell)	Serum and Plasma	Increased	Elevation associated with more severe pain (VAS) and worse FIQ scores.	[66]
	Calgranulin A/C *	Saliva	Increased	Potentially linked to inflammatory or oxidative stress processes in the FM	[42]
	Serotransferrin *	Serum	Increased	No correlation with severity	[67]
		Saliva			[43]
**Other relevant Biomolecules**	Ascorbic Acid	Serum	Decrease	Significantly decreased in FM patients compared with healthy controls	[68]
	Cortisol *	Saliva	Altered	Association between high levels of cortisol in early stages of the pathology that correspond to peaks in pain, stress and depression.	[69,70,71]
	TAC (Total Antioxidant Capacity)	Serum	Decreased	Low cortisol levels are associated with the duration of the disease, and may be the cause of chronic adaptation to stress in FM patients	[68]
	OSI (Oxidative Stress Index)	Serum	Increased	Calculated from the TOS/TAC ratio; a marker of oxidative imbalance in FMS	[68]
	Uric Acid/Creatine acid	Blood	Increased	Ratio is positively associated with pain intensity and disease severity	[72]

* Biosensors using saliva as the sample. Abbreviations: 3-MH (3.metil histidine), 5-HT (serotonin), 5-MH (5.metil histidine), α-APA (α-aminopeptidase), AAA (aromatic amino acid), Ala (alanine), Arg (arginine), Asn (asparagine), Cit (citrulline), CCL2 (C-C motif ligand 2), CSF (Cerebrospinal fluid), CXCL (Chemokine CXC Ligand), FIQ (Fibromyalgia Impact Questionnaire), FM (fibromyalgia), Gln (glutamine), Hyp (hydroxyproline), Ig (immunoglobulin), IL (interleukin), Lys (lysine), Met (methionine), Orn (ornithine), OSI (Oxidative Stress Index), PGAM-1 (Phosphoglycerate mutase 1), Pro (proline), Ser (serine), SP (Substance P), TAC (Total Antioxidant Capacity), TALDO1 (Transaldolase 1), TNF (tumor necrosis factor), Thr (threonine).

Together, these molecules underscore the intricate interplay between neuroendocrine, metabolic, and oxidative pathways. While their individual diagnostic value may be modest, their integration into multimodal biomarker panels significantly enhances the capacity to objectively monitor disease activity, symptom fluctuations, and therapeutic response.

### 2.7. Saliva as a Strategic Matrix for FM Monitoring

The transition toward objective disease monitoring in FM necessitates the use of non-invasive biological fluids, among which saliva is increasingly recognized as a strategic matrix. The use of saliva offers distinct advantages for longitudinal tracking by allowing for stress-free, repeated sampling, thereby avoiding the confounding effects of needle-stick-induced stress—a factor particularly relevant when measuring HPA axis markers such as cortisol. However, the adoption of salivary biomarkers into clinical practice faces specific methodological challenges; salivary concentrations are highly dependent on collection protocols (unstimulated vs. stimulated saliva) and circadian rhythms, and reference ranges remain less standardized than those in serum or plasma. Furthermore, many cytokines and neuropeptides circulate at ultra-low concentrations, often close to current assay detection limits, requiring analytical methods with ultra-low sensitivity—such as ELISA or LC–MS/MS—and careful methodological alignment for inter-study comparisons. While most traditional FM biomarkers, including certain antibodies, serine, ascorbic acid, and specific amino acids, are not yet fully validated in this matrix, several proteins and enzymes—notably α-amylase, cortisol, PGAM1, transaldolase, and calgranulins—show great promise. Nonetheless, it must be noted that markers like α-enolase, PGAM-1, and IgG anti-satellite glial cell antibodies currently lack validated clinical reference ranges and are used exclusively in research contexts. Despite the pronounced diurnal and stress-related variability of hormonal and autonomic markers, ongoing research is continuously expanding the salivary biomarker panel, representing a transformative path toward improved diagnosis, phenotype stratification, and personalized care for FM patients [30,42,43,44,47,49].

## 3. Electrochemical Biosensors: A Translational Platform for Fibromyalgia Biomarker Monitoring

### 3.1. Current Analytical Landscape and Rationale for Electrochemical Biosensors in FM

Current research on FM has explored multiple analytical approaches to identify potential biomarkers, ranging from classical immunoassays to advanced omics-based methods and spectroscopic techniques.

Traditional methods, such as Enzyme-Linked Immunosorbent Assays (ELISA), are frequently employed to quantify soluble mediators, including cytokines, chemokines, autoantibodies, and other immune-related proteins, leveraging high antibody specificity [73]. These efforts have been expanded by proteomic studies, which analyze the comprehensive protein profile in FM patients; a recent systematic review identified over 3300 proteins, with approximately 145 exhibiting significant alterations compared to healthy controls, highlighting differences in protein expression that may serve as potential biomarkers [39].

Similarly, metabolomic analyses of plasma or serum have revealed changes in small molecules associated with neurotransmission and amino acid metabolism, such as glutamate and serine, which are linked to pain, fatigue, and oxidative stress in FM. They highlight that gut microbiome analysis combined with serum metabolomics can shed new light onto the pathogenesis of FM [74]. Furthermore, spectroscopic approaches, including Fourier-transform infrared (FT-IR) spectroscopy combined with chemometric techniques, have demonstrated the capacity to discriminate biochemical profiles of FM patients from healthy controls or individuals with other rheumatic conditions [75].

Despite these analytical advances, the marked heterogeneity of FM and the temporal variability of its clinical manifestations pose significant challenges for conventional biomarker detection. Methods relying on peripheral blood collection via venipuncture and subsequent laboratory processing often provide only single-point measurements (spot measurements), which may not accurately capture the dynamic, fluctuating nature of the syndrome or differentiate meaningful signals from noise. In this context, there is a clear need for technologies capable of continuous or repeated monitoring under minimally invasive conditions.

In this sense, electrochemical biosensors have emerged as a particularly promising strategy to overcome these critical limitations. Their ability to operate with extremely small sample volumes and utilize non-invasive biofluids, such as saliva, sweat, or tears, substantially reduces patient inconvenience and procedural demands, which is crucial for chronic conditions that require repeated, high-frequency measurements. Crucially, these platforms can be readily adapted through miniaturization and integration into portable or wearable formats, enabling Point-of-Care (PoC) deployment and high-frequency longitudinal assessment in real-world settings. Rapid physiological fluctuations often remain undetected by conventional laboratory assays. In contrast, the inherent speed of electrochemical transduction provides fast analytical responses, enabling near real-time feedback for both patients and clinicians. Moreover, the use of materials and fabrication techniques compatible with scalable and low-cost manufacturing, such as printed electrodes, paper-based electrodes, and functional nanomaterials, greatly enhances their translational potential, facilitating mass production and broad clinical or commercial deployment [76,77,78]. In addition, the inherent high sensitivity and selectivity achievable with modern electrochemical sensor design align perfectly with the clinical needs of FM, supporting earlier and more precise characterization of FM-related biological alterations and improving patient monitoring and clinical management.

An important translational consideration when evaluating candidate biosensors is whether the analytical sensitivity of current platforms is compatible with the physiological concentration ranges of biomarkers in clinically accessible fluids such as saliva. Many salivary biomarkers relevant to FM occur at low concentrations, typically within the pg/mL to ng/mL range. For example, salivary 5-HT concentrations have been reported between approximately 0.32 and 9.62 ng/mL [79], while cortisol levels commonly range from 0.2 to 5.8 ng/mL depending on circadian timing [80]. Inflammatory cytokines such as IL-1β are often detected in the tens of pg/mL range in saliva [81]. Consequently, biosensors intended for non-invasive monitoring must achieve detection limits that fall well below these physiological levels to ensure reliable quantification. Encouragingly, several nanomaterial-enhanced electrochemical biosensors reported in the recent literature demonstrate limits of detection within or below these concentration ranges, suggesting that analytical sensitivity may be sufficient for translational applications. However, additional challenges remain, including matrix effects, signal stability, and inter-individual variability in biomarker concentrations, which must be addressed before these platforms can be implemented in routine clinical monitoring. Importantly, the clinical interpretation of salivary biomarkers also depends on the degree of correlation with systemic concentrations. While certain markers, such as cortisol, show strong agreement with circulating free hormone levels, the relationship between salivary and blood concentrations for inflammatory cytokines and neurotransmitters remains more variable and context dependent. This variability highlights the need for careful clinical validation when translating non-invasive biosensing approaches into diagnostic or monitoring tools for FM.

### 3.2. Electrochemical Biosensing of Relevant FM Biomarkers: Proof-of-Concept and Opportunities

#### 3.2.1. The Current Translational Gap in FM Biosensing

Different studies have applied electrochemical approaches to assess autonomic and sudomotor dysfunction in FM. For example, electrochemical skin conductance (ESC) combined with quantitative sensory testing has been used to characterize physiological alterations in FM [82]. This approach revealed altered sudomotor function and impaired autonomic responses, indicating that FM patients exhibit dysregulated sympathetic activity. Also, Reyes del Paso et al. applied electrodermal measurements to assess sympathetic nervous system activity at rest in women with FM. Their findings indicate reduced tonic skin conductance and blunted reactivity, reflecting diminished sympathetic activity and impaired autonomic regulation, consistent with small fiber neuropathy in FM [83]. Importantly, while these approaches utilize electrochemical measurements, they do not detect specific molecular analytes and therefore cannot be considered biosensors. By definition, a biosensor requires a specific biorecognition element, such as an antibody, aptamer, or enzyme, that interacts with a molecular target, coupled to a transducer that converts this interaction into a quantifiable signal. Nevertheless, these studies highlight the potential of electrochemical methods to capture dynamic physiological changes in FM, underscoring the need for complementary molecular biosensing platforms that can provide highly specific, real-time insights into the biochemical underpinnings of the syndrome.

Consequently, despite the growing clinical interest in monitoring candidate FM biomarkers in non-invasive biofluids, no electrochemical biosensor employing a biorecognition element has yet been reported for the direct analysis of patient samples in FM. This highlights a significant translational gap. However, recent advances in wearable and non-invasive electrochemical biosensor technologies for healthcare demonstrate the technical feasibility of these platforms, offering flexible, low-cost, scalable, and highly sensitive detection of molecular biomarkers in non-invasive biofluids, with promising applications reported in contexts such as metabolic disorders, stress monitoring, cardiovascular disease, and cancer [84,85]. The successful development of these platforms establishes a clear proof-of-concept for adapting similar electrochemical approaches to the specific biomarkers relevant to FM. In the following section, the development of electrochemical biosensors for the detection of biomarkers relevant to FM will be explored, categorizing them according to their chemical and biological nature. Special emphasis will be placed on biosensors designed for non-invasive matrices, as they represent the most promising frontier for achieving continuous, pain-free, and objective monitoring in clinical and PoC settings (see Table 2).

In the field of electrochemical biosensors for biomarker detection, diverse molecular recognition strategies have been developed, each with distinct analytical characteristics that determine their suitability based on the biomarker type, expected concentration, and complexity of the biological matrix. Selecting the appropriate recognition element is critical to achieving sensitivity, selectivity, and analytical stability in real samples.

Aptasensors are distinguished by high sensitivity (nM–pM range) and exceptional specificity derived from the three-dimensional folding of the aptamer, alongside superior chemical and thermal stability compared to conventional antibodies and facile functionalization on nanomaterials. These properties make them particularly suitable for detecting biomarkers such as 5-HT, DA, and IL-6, even in complex matrices like saliva or sweat [86,87]. However, matrix components such as proteins can interfere with signal transduction, often necessitating antifouling strategies or surface modifications to maintain selectivity and stability [88,89,90,91,92,93].

Enzymatic biosensors offer intrinsic specificity toward their substrates and signal amplification via catalytic reactions, enabling rapid, real-time monitoring. They are well-suited for analytes such as catecholamines, α-amylase, and glycine, where enzymatic activity provides a robust detection mechanism [94,95]. Their performance, however, is sensitive to enzyme activity, pH, temperature, and matrix effects, which can compromise reproducibility in biological fluids.

Immunosensors, based on antibody–antigen affinity, achieve ultra-high sensitivities, making them ideal for trace-level biomarkers such as cytokines (IL-6, TNF-α, IL-10) [87,96,97] and chemokines (CXCL9/10) [98]. They maintain high specificity in complex matrices such as plasma, serum, or urine but face operational limitations including biofouling, bioreceptor instability, long incubation times, and typically single-use configurations [90].

Molecularly imprinted polymer (MIP)-based sensors combine chemical robustness, long-term stability, and high selectivity, offering an attractive alternative for continuous or long-term monitoring. These systems are suitable for biomarkers such as cortisol and acetylcholine, with selectivity depending on polymer design and imprinting conditions to ensure effective recognition in complex samples [88,89].

Finally, direct non-enzymatic electrochemical sensors provide structural simplicity and resilience under adverse conditions. They are applicable to biomarkers such as NE and ascorbic acid, where the absence of bioreceptors minimizes denaturation. Nevertheless, specificity can be compromised by electroactive interferents, and signal drift may require surface modification or correction strategies to ensure analytical reliability.

Collectively, despite advances in sensitivity, device miniaturization, and integration with nanomaterials, a central challenge remains achieving simultaneously high selectivity, operational stability, and reliable analytical performance in real biological matrices. The choice of biosensing strategy should consider not only the expected biomarker concentration but also sample composition, potential interferents, and the specific requirements of the intended application, particularly for non-invasive and continuous monitoring platforms.

It should also be noted that many of the sensing platforms summarized in Table 2 have primarily been validated in controlled laboratory conditions or spiked samples, and further validation using authentic clinical specimens remains necessary.

#### 3.2.2. Biosensors for Neurotransmitters and Neuromodulators Detection

Considering the strong link between FM and neurochemical imbalances, electrochemical platforms for monitoring neurotransmitters and inflammatory mediators are highly relevant. Electrochemical sensors, including both biosensors that employ specific biorecognition elements and those detecting electrochemical changes without incorporated biomolecules, have demonstrated the capability to detect small neurotransmitters in non-invasive or minimally invasive biofluids such as sweat or interstitial fluid.

**Table 2:** Analytical properties of electrochemical biosensors for the detection of FM-related biomarkers in non-invasive biofluids.

	Biomarker	Biofluid	LOD	Linear Range	Electrochemical Technique	Immobilization System	Bioreceptor	Ref.
Neurotransitters	5-HT, DA, EN	Human Sweat	0.33 nM (5-HT), 0.18 nM (DA); 0.27 nM (EN)	1 nM–10 µM	SWV	AuNPs-modified CuMOF@InMOF heterostructure	Thiolated DNA Aptamer	[86]
	DA/EN	Artificial sweat	10 nM	Up to 100 nM	Amperometry	Site-specific enzyme immobilization	Enzyme (CueO)	[99]
	NE	Tear fluid	Not reported	Not reported	Amperometry	Electropolymerized polytyramine	None (direct electrochemical detection)	[100]
	ACh	Human Saliva	0.012 nM	0.1–10 nM	CV	PVC	MIP	[101]
Amino acids	Glycine	Human sweat	Not specified	25–500 µM	Amperometry	chitosan matrix with Nafion^®^	Enzyme-based catalyst (quinoprotein)	[94]
enzymes	α-Amylase	Saliva	0.041 U/mL	0.5–280 U/mL	DPV	MWCNT/β-cyclodextrin/starch	Enzymatic substrate recognition	[95]
	α-Amylase	Saliva	0.00196 U/mL	Not reported	EIS	ZnO/Cu-doped nanocrystals	Antibody	[102]
Cytokines and chemokines	IL-8	Saliva	Not reported	Not reported	Amperometry	magnetic microparticles	Antibody	[103]
	IL-10	Saliva	0.02 pg/mL	Not reported	IMFET	Antibody functionalization on the transistor-sensing gate	Antibody	[96]
	CXCL9 and CXCL10	Unprocessed urine	1–9 pg/mL	Not reported	EIS	Mxene–BSA hydrogel	Antibody	[98]
	IL-6	Saliva/urine	0.39 pg/mL	1.75–500 pg/mL	Amperometry	magnetic microparticles	Antibody	[104]
	TNF-α	Saliva	3.1 pg/mL	Not reported	EIS	Carboxyl-diazonium functionalization of gold microelectrode array	Antibody	[105]
	IL-6/TNF-α	Saliva and sweat	1.6 pg/mL	5–5000 pg/mL	SWV	Au/Pt NPs	Aptamers	[87]
	IL6, IL8, IL10, TNF-α	Sweat	0.2 pg/mL	0.2–200 pg/mL.	Amperometry	self-assembled monolayer (SAM)	Antibody	[106]
Other relevant molecules	Cortisol	Saliva	0.048 pg/mL	0.10–10,000 pg/mL	DPV	graphene-modified thread electrode	MIP	[107]

Abbreviations: 5-HT (serotonin), AuNPs (gold nanoparticles), BSA (bovine serum albumin), CV (cyclic voltammetry), CueO (copper efflux oxidase), DA (dopamine), DPV (differential pulse voltammetry), EIS (electrochemical impedance spectroscopy), EN (epinephrine), IMFET (ion-modulated field-effect transistor), LOD (limit of detection), MIP (molecularly imprinted polymer), MOF (metal–organic framework Mxene (material 2D tipo carburo/nitruro metálico), PVC (polyvinyl chloride), SAM (self-assembled monolayer).

Neuropeptides such as SP, which is consistently elevated in the central nervous system of FM patients, present a significant challenge for salivary detection due to their high molecular weight and extremely low concentrations in oral fluids compared to serum. Consequently, current state-of-the-art electrochemical platforms are still primarily validated in blood-derived matrices. For instance, a miniaturized microfluidic immunochip was developed for SP quantification in serum, utilizing an integrated electrochemical electrode for antigen–antibody detection. This device achieved a LOD of 15.4 pg/mL using only 5 µL of sample in 10 min [108]. While this represents a major step toward PoC testing, the translation to saliva would require further signal amplification strategies to overcome the matrix effects and lower analyte abundance.

Similarly, while 5-HT is critical in FM pathophysiology, its electrochemical detection in saliva is often hampered due to extremely low physiological concentrations and overlapping oxidation potential with common interferents like ascorbic acid and uric acid, which are abundant in the oral cavity. While biosensors for 5-HT in serum or model solutions exist [109,110,111], robust detection directly in non-invasive fluids remains limited. For example, a recently reported wearable electrochemical aptasensor based on a metal–organic framework (MOF-on-MOF) heterostructure enabled simultaneous real-time detection of multiple neurotransmitters, including 5-HT, DA, and EN, in human sweat collected from healthy volunteers during physical activity. The flexible sensor integrates specific nucleic acid aptamers for each neurotransmitter on a microfluidic patch that adheres to the skin, facilitating non-invasive sweat sampling and continuous analysis. Using square wave voltammetry, the device achieved low limits of detection in the sub-nanomolar range (e.g., ~0.33 nM for 5-HT) and successfully quantified neurotransmitter concentrations in sweat [86].

Other essential catecholamines, such as EN and NE, have seen progress using enzymatic and biomimetic approaches. In this context, an enzymatic biosensor using site-specifically immobilized copper efflux oxidase (CueO) successfully monitored DA and EN in artificial sweat with a LOD of 10 nM [99]. A flexible, rolled, thick film amperometric sensor was developed for minimally invasive monitoring of biomarkers in tear fluid, demonstrating sensitive in vitro responses to NE. The device, designed for insertion into the lacrimal canaliculus, incorporates an electropolymerized polytyramine layer to reduce interference from common electroactive species. While primarily tested in controlled conditions, this study highlights the potential of electrochemical sensors for NE detection in non-invasive biofluids [100].

Numerous electrochemical biosensors for the determination of ACh have been reported in the literature. Most of the applied enzymes rely on the immobilization of acetylcholinesterase (AChE) or combined AChE–choline oxidase (ChO) enzymatic systems and have mainly been applied to biological matrices such as blood, plasma, serum, and tissue samples [112,113]. However, direct detection of ACh in human saliva remains scarcely explored. In this context, a recently developed molecularly imprinted polymer (MIP)–based electrochemical sensor represents a significant advance toward non-invasive analysis. The MIP was synthesized in the presence of ACh as a template, blended with a PVC binder, and coated onto a glassy carbon electrode. Detection was achieved using a ferro/ferricyanide redox probe and cyclic voltammetry, where ACh binding to the imprinted cavities resulted in a decrease in current response. The sensor exhibited a linear detection range of 0.1–10 nM, a limit of detection of 0.9 nM in buffer, and high selectivity against interfering neurotransmitters such as glutamate and GABA. Importantly, the sensor was successfully validated using real saliva samples, achieving an ultra-low detection limit of 0.012 nM and quantifying an ACh concentration of approximately 0.95 nM [101].

#### 3.2.3. Biosensing Approach for Amino Acids Detection

Electrochemical biosensors have demonstrated high sensitivity and selectivity for small-molecule analytes, providing a robust technological foundation for monitoring physiologically relevant metabolites. The maturity of these platforms is exemplified by their widespread application in wearable, sweat-based systems for measuring metabolic intermediates such as glucose and lactate. Recent advancements include human sweat glucose sensors based on carbon black nanoparticles [114], biofuel cell-powered devices for lactate monitoring [115], and innovative on-body platforms employing paper-based microfluidics with 3D-printed flexible electrodes [116]. While these technologies do not target FM-specific biomarkers directly, they validate the translational potential and technical readiness of electrochemical sensing for non-invasive clinical applications [117].

Although a variety of electrochemical biosensors have been developed for amino acid neurotransmitters such as glutamate, glycine, and d-serine, few have been successfully translated to truly non-invasive biofluids like saliva or sweat. Glutamate oxidase (GluOx)–based amperometric sensors remain the gold standard for glutamate due to their high selectivity in controlled matrices, but there are no well-validated reports of glutamate biosensors operating directly in human saliva or other non-invasive fluids to date, highlighting a significant translational gap. However, as highlighted in recent comprehensive reviews, the transition to real-world applications requires addressing challenges such as enzyme instability and the detection of subtle physiological fluctuations. Strategies including surface passivation, on-chip signal amplification, and probe miniaturization are being developed to enhance the temporal resolution and sensitivity required for clinical monitoring [118].

In the case of glycine, an amperometric biosensor based on a quinoprotein catalyst and Prussian blue mediator was developed for the quantitative determination of glycine in multiple biological fluids, including blood/serum, urine, and notably human sweat. The sensor features a chitosan-encapsulated quinoprotein with an outer Nafion^®^ layer to suppress interference from compounds like ascorbic acid. It exhibits a fast response (<7 s), good reproducibility and stability, and a wide linear range (25–500 μM) covering physiological glycine levels. Importantly, the sensor was validated in real samples, including sweat from healthy individuals, demonstrating results consistent (<9% discrepancy) with a commercial fluorescence kit [94].

Serine, an essential NMDA receptor co-agonist, has been quantified in vivo using enzymatic amperometric biosensors. By incorporating poly(meta-phenylenediamine) permselective layers, these sensors achieve high selectivity and rapid response times, enabling localized detection of d-serine release with fine spatial and temporal resolution [119]. Although these devices have not yet been adapted for saliva or other non-invasive matrices, they represent a technologically relevant foundation for future non-invasive monitoring platforms. The main challenges to translating d-serine detection to saliva include the presence of complex organic interferents and the variable pH of the oral matrix, which necessitate stable enzymatic interfaces and interference-suppressing layers to enable reliable measurement of the excitatory–inhibitory amino acid balance in non-invasive samples.

#### 3.2.4. Biosensor-Based Detection of Enzymes

Enzymes play pivotal roles in energy metabolism, redox balance, and stress response pathways, many of which are dysregulated in FM. Proteomic analyses have identified several enzymes, including α-amylase, PGAM1, TALDO1, and α-enolase, as differentially expressed in biological fluids of FM patients, highlighting their potential as biomarkers for disease monitoring. The following sections summarize current electrochemical biosensor technologies where available, as well as other analytical approaches developed for these enzymes in the absence of dedicated biosensors.

Recent advances in salivary α-amylase (α-ALS) biosensing highlight two complementary electrochemical strategies: nanointerface-enhanced enzymatic sensing and nanocrystal-based immunodetection. On one hand, a MWCNT/β-cyclodextrin/starch-modified screen-printed electrode using a ferrocene redox-switch efficiently converts the catalytic hydrolysis of starch by α-ALS into an amplified electrochemical inhibition signal, yielding an exceptionally wide linear range (0.5–280 U/mL) and a low LOD (0.041 U/mL). Importantly, the sensor demonstrates strong selectivity against common salivary interferents and exhibits excellent accuracy in both artificial saliva (97–103% recovery) and real clinical samples from patients with dental caries [95]. On the other hand, a ZnO/Cu-doped nanocrystal immunosensor employs antibodies as the biorecognition element and detects α-ALS binding via electrochemical impedance spectroscopy, where formation of the immunocomplex increases charge-transfer resistance. Although Cu incorporation enhances conductivity and antibody loading, its effect depends strongly on the assembly order, underscoring the need for careful interface engineering to avoid nonspecific interactions. A detection limit of 0.00196 U·mL^−1^ was obtained. Together, these platforms illustrate how nanostructured materials (whether acting as catalytic enhancers or as support for antibody recognition), can markedly improve sensitivity and selectivity in salivary α-amylase detection, reinforcing its promise as a clinically relevant biomarker.

Proteomic analyses have identified PGAM1 among the proteins differentially expressed in biological fluids of patients with FM, implicating altered glycolytic and energy metabolism pathways in the syndrome’s complex pathophysiology. Although several sandwich ELISA kits exist for quantitative measurement of PGAM1 in biological samples (e.g., human PGAM1 ELISA kits with sensitivities in the sub-nanogram per milliliter range, biomatik.com reference EKF57684-PI), to date, no biosensor has been developed specifically for PGAM1 detection, highlighting an unmet need for novel biosensing platforms targeting this enzyme in clinical and research settings [120]

Similarly, TALDO1, a key enzyme in the pentose phosphate pathway, has been identified as differentially expressed in proteomic analyses of patients with FM, suggesting potential alterations in cellular redox balance and metabolic flux [39]. As with PGAM1, its quantification is currently possible using commercial ELISA kits, such as the *GENLISA™ Human Transaldolase* ELISA Kit (sensitivity 0.05 ng/mL; Krishgen Biosystems, Mumbai, India) and the *FineTest^®^ Human Transaldolase 1* ELISA Kit (sensitivity 0.094 ng/mL; FN-Test, Wuhan Fine Biotech, Wuhan, China), with no electrochemical biosensor developed to date for direct detection [121,122].

**Figure 1:**
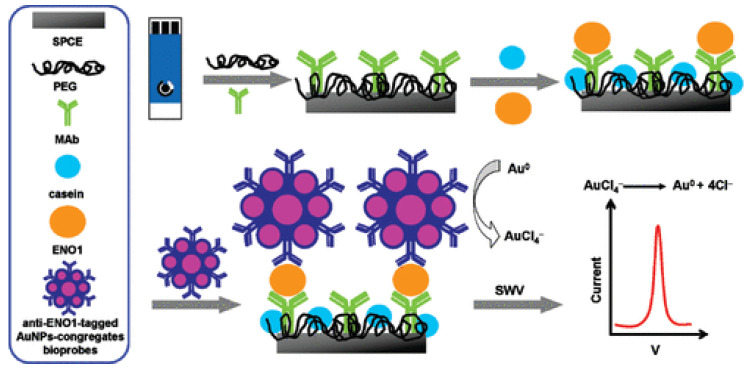
Schematic representation of the operation of the electrochemical immunosensor for the detection of ENO1. Reproduced with permission from [123].

Alpha-enolase (ENO1), a glycolytic enzyme involved in energy metabolism, has been implicated in various pathophysiological conditions, including cancer and potentially FM. To date, no electrochemical biosensors have been reported for the detection of alpha-enolase (ENO1) in saliva or other non-invasive human fluids. However, highly sensitive platforms have been successfully validated in human serum, demonstrating excellent analytical performance. For example, Ho et al. (2010) developed an electrochemical immunosensor for ENO1 in a sandwich format, using anti-ENO1 antibodies immobilized on a gold nanoparticle-modified carbon electrode and secondary antibody-conjugated AuNPs for signal amplification [123]. Detection was performed via square wave voltammetry (SWV), achieving an ultralow limit of detection (~11.9 fg/mL) and a linear response range from 10^−12^ to 10^−8^ g/mL, validated in human serum as a biomarker of lung cancer (Figure 1).

As discussed previously, PoC devices are essential for performing early, sensitive, and specific detection of different biomarkers at a lower cost. Label-free biosensors may represent a suitable option for this type of device. In this context, Aydın et al. (2020) reported a label-free impedimetric immunosensor on epoxy-substituted polypyrrole-modified ITO electrodes to detect Neuron-Specific Enolase (NSE/γ-enolase), an isoform predominantly expressed in neurons and neuroendocrine tissues. Using anti-NSE antibodies as the biorecognition element, the sensor demonstrated a linear range of 0.02–7.5 pg/mL and a limit of detection of 6.1 fg/mL in human serum [124].

#### 3.2.5. Biosensor-Based Detection of Cytokines and Chemokines

Inflammatory mediators such as cytokines (e.g., IL-10, IL-6, IL-8, TNF-α) and chemokines (e.g., CCL2, CCL5, CXCL6, CXCL10) are implicated in FM pathophysiology. Although electrochemical biosensors for these specific molecules have not yet been applied directly to FM patient samples, high-sensitivity platforms have been successfully developed for cytokine and chemokine detection in other clinical contexts, capable of simultaneous quantification of multiple inflammatory markers in small volumes of saliva, plasma, or interstitial fluid [125].

For instance, an electrochemical immunosensor has been developed for the detection of salivary IL-8 at both the protein and mRNA levels, demonstrating direct quantification in undiluted human saliva with high sensitivity using dual amperometric magnetobiosensor platforms. Although highly innovative, this system measures a single biomarker in two formats, rather than enabling true multiplexed detection of multiple distinct analytes, and was originally applied for oral-cancer biomarker analysis [103].

Additionally, a salivary IL-10 detection strategy has been implemented using an easily integrable Lab-on-Chip and PoC Ion-Modulated Field-Effect Transistor (IMFET) platform. The developed IMFET exhibited a LOD of 0.02 pg/mL in real saliva. And good selectivity toward IL-10 in the presence of other biomarkers such as cortisol, or TNF-α [96]. Moreover, emerging work aiming to develop electrochemical biosensors for IL-10 detection in complex biological media (e.g., plasma) demonstrates sensitive immunosensing of IL-10 within the low pg/mL range using antibody-modified microelectrode surfaces, providing a clear roadmap for adapting such strategies to saliva [126].

**Figure 2:**
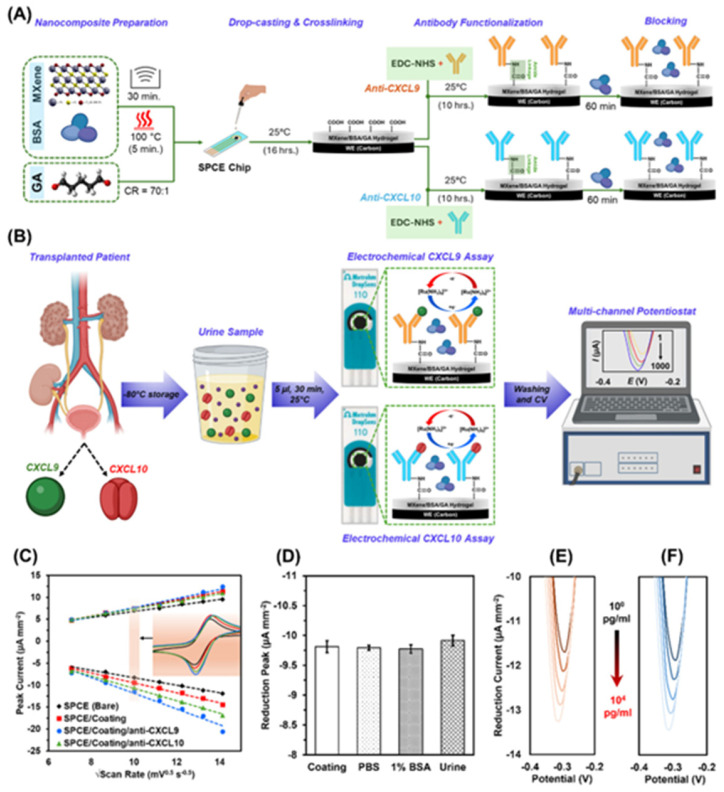
(**A**) Fabrication of CXCL9 and CXCL10 assays using SPCE modified with MXene/BSA/GA nanocomposite, (**B**) detection protocol from urine collection to signal extraction, (**C**) scan rate–dependent peak currents for bare, nanocomposite-coated, and antibody-functionalized SPCEs (inset: CV at 100 mV s^−1^), (**D**) reduction peak currents after 24 h incubation in PBS, 1% BSA, and urine; no significant differences observed, indicating no electrode passivation (n = 3), (**E**,**F**) reduction current responses for CXCL9 and CXCL10 detection (1–10^4^ pg mL^−1^) in urine, showing increasing signal with concentration. Reproduced with permission from [98].

Electrochemical biosensors have also been applied to the detection of chemokines in clinical biofluids, highlighting their potential for rapid, non-invasive monitoring. For instance, a label-free electrochemical immunosensor was developed for the simultaneous quantification of urinary CXCL9 and CXCL10, achieving sensitivity in the range of 1–9 pg/mL from only 5 µL of unprocessed urine in 15 min, compared to 24–72 h required for conventional ELISA or biopsy (Figure 2). The system employs screen-printed carbon electrodes modified with a Mxene–BSA hydrogel, providing high conductivity, anti-fouling properties, and signal stability. The biorecognition element consists of specific antibodies immobilized on the electrode surface, and detection relies on the selective binding of the target chemokines, generating measurable electrochemical signals without enzymatic catalysis [98]. 

Another example reported an anti-CCL5 immunosensor capable of quantifying CCL5 at clinically relevant levels and discriminating patients with multiple sclerosis from healthy individuals [127]. Despite these advances, direct electrochemical detection of chemokines in human saliva has not yet been demonstrated, reflecting the analytical challenges posed by complex biofluids and highlighting an important opportunity for future biosensor development. In the case of CCL2, reviews of current biosensing strategies emphasize ongoing efforts to engineer affinity-based electrochemical platforms for selective chemokine quantification, underscoring the significant potential for future salivary CCL2 monitoring [97].

IL-6 is a key mediator in chronic inflammatory and autoimmune processes and is involved in the modulation of pain and fatigue. Its determination is of interest in FM, which is associated with low-grade inflammation, although it is not a specific diagnostic biomarker. A highly sensitive magnetoimmunosensor for IL-6 detection was developed using magnetic microparticles and poly-HRP signal amplification. The sensor showed an improved signal-to-blank ratio, a linear range of 1.75–500 pg/mL, and a low detection limit of 0.39 pg/mL. It exhibited excellent stability and selectivity. IL-6 was successfully measured in urine and saliva samples with results comparable to ELISA.

This approach enables IL-6 determination in non-invasive samples and may be useful for FM studies [104]. Another approach, although currently validated in serum samples, is the use of label-free architecture, which serves as a critical proof-of-concept for the future of rapid, low-cost FM screening. In this context, a label-free electrochemical inmunosensor based on a sandwich format was developed for IL-6 detection in patients affected by psoriasis. The immunosensor exhibits a wide linear range (2–250 pg/mL), a low limit of detection (0.78 pg/mL), and good reproducibility (RSD < 7%). Moreover, it showed a strong correlation with ELISA in real samples, with the added advantages of faster analysis and easier handling [128].

For the determination of TNF-α, various electrochemical biosensors have been developed. TNF-α is a key cytokine in inflammation and immune system regulation, playing a critical role in the development and progression of chronic and autoimmune diseases. Recent progress in electrochemical biosensing has established saliva, tears, and urine as viable non-invasive matrices for TNF-α monitoring. Research highlights a transition toward both sandwich and label-free architectures, where the integration of nanostructured materials is pivotal for enhancing sensitivity and signal-to-noise ratios. Among these platforms, salivary immunosensors are particularly prominent due to the matrix’s stability and clinical relevance. Most reported devices achieve detection limits in the pg/mL range, meeting the requirements for early systemic inflammation and cardiovascular monitoring [129]. For example, an integrated electrochemical platform featuring eight gold microelectrodes functionalized with carboxyl diazonium was developed for the highly sensitive detection of TNF-α in human saliva using EIS, with a detection limit of 3.1 pg/mL [105].

To date, there are no reported electrochemical biosensors for the direct detection of CXCL6 in saliva or other non-invasive human biofluids, despite its emerging relevance as a biomarker in conditions involving immune dysregulation and chronic pain syndromes such as f FM. Current methods for quantifying CXCL6 rely on immunoassay-based platforms; for example, sandwich ELISA kits such as the GENLISA™ Human CXCL6 ELISA allow quantitative measurement of CXCL6 in serum, plasma, and cell culture supernatants with sensitivities around ~15.5 pg/mL, providing reliable protein quantification in research settings [130]. Similarly, other commercial kits like the Human CXCL6/GCP-2 Quantikine ELISA Kit offer sensitive detection in serum and plasma with limits of detection in the single-digit pg/mL range, enabling robust analysis of circulating chemokine levels [131].

#### 3.2.6. Biosensing Approach for Non-Enzymatic Proteins Detection

Inflammation- and stress-related proteins such as calgranulin A (S100A8) and serotransferrin have been increasingly recognized as potential biomarkers in FM. Calgranulin A (S100A8), a calcium-binding protein of the S100 family, is involved in inflammatory processes, immune response modulation, and oxidative stress, and has been found elevated in saliva and serum of patients with FM, suggesting its potential role in disease pathophysiology. To date, no electrochemical biosensors have been reported for direct detection of S100A8/calgranulin A. Quantitative measurement is feasible using commercial ELISA kits, such as the BioAssay™ S100A8 ELISA Kit (<10 pg/mL sensitivity; applicable to serum, plasma, and saliva; BioCompare) and the R&D Systems Human S100A8 ELISA Kit (DY8058) (~5 pg/mL sensitivity; serum and plasma; R&D Systems, Minneapolis, MN, USA) and the S100A8(Protein S100-A8) BioAssayTM ELISA Kit (Human,358653), (United States Biological, Salem, MA, USA) [132]. Serotransferrin (transferrin), a plasma glycoprotein responsible for iron transport and homeostasis, has been reported as differentially expressed in patients with FM, suggesting alterations in iron metabolism and oxidative stress pathways associated with the disease. Quantitative measurement is feasible using commercial ELISA kits, such as the Abcam Human Transferrin ELISA Kit (ab108902) (~0.36 ng/mL sensitivity; serum and plasma; Abcam) and the Cloud-Clone Human Transferrin ELISA Kit (SEA797Hu) (~0.17 ng/mL sensitivity; serum, plasma, and saliva; Cloud-Clone), the latter allowing analysis in non-invasive saliva samples. To date, no electrochemical biosensors have been reported for direct detection of serotransferrin [133].

#### 3.2.7. Sensing Strategies for Other Relevant Biomolecules

**Figure 3:**
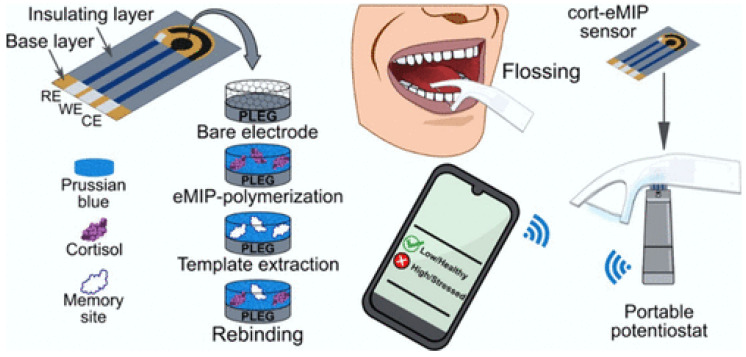
Proposed saliva-sensing dental floss use case with the embedded cort-eMIP biosensor. Reproduced with permission from [107].

Hormonal indicators linked to stress-axis dysregulation, particularly salivary cortisol, represent valuable targets for monitoring the chronic stress commonly observed in FM. Several electrochemical biosensors have been reported for the detection of cortisol in human biofluids [134], incorporating diverse recognition elements such as antibodies, MIPs, and aptamers. Special emphasis has been placed on understanding how these recognition strategies influence sensor selectivity, sensitivity, and overall analytical performance. Recently, Sharma et al. (2025) reported a dental-floss-based electrochemical sensor for salivary cortisol detection (Figure 3). The device integrates MIPs as synthetic recognition elements with thread-based microfluidics on a high-surface-area graphene electrode. This configuration enabled highly selective and sensitive detection across a broad dynamic range (0.10–10,000 pg/mL), achieving an ultralow limit of detection of 0.048 pg/mL. The analytical performance showed excellent agreement with conventional ELISA measurements [107].

Metabolites associated with FM that are involved in oxidative stress and inflammation, such as ascorbic acid (AA) and uric acid (UA), are important biomarkers in the study of the disease. Recent reviews highlight the effectiveness of electrochemical biosensors for detecting uric acid in non-invasive samples [135,136]. Furthermore, non-enzymatic electrochemical sensors based on nanomaterials (including noble metals, bimetallic alloys, conducting polymers, and carbon-based nanostructures) provide the high sensitivity, selectivity, and stability required for AA analysis. These technologies enable precise monitoring of ascorbic acid levels, supporting deeper research into its correlation with FM symptoms and the development of targeted therapeutic interventions [137].

#### 3.2.8. The Next-Generation Strategy: Multiplexed Biosensing

The complex and heterogeneous nature of FM necessitates the monitoring of a panel of biomarkers, including neurochemical, hormonal, and inflammatory mediators, rather than focusing on a single analyte. In this context, the development of multiplexed electrochemical biosensors offers a highly promising strategy for comprehensive, minimally invasive biomarker monitoring in FM, as it reduces the required sample volume, shortens analysis time, enables a more holistic assessment of the patient’s biochemical profile, and allows for the correlation of multiple biomarker fluctuations with symptoms, stress, or treatment response [138]. Although no multiplexed platforms have yet been validated in FM patients, they have shown promising performance in other clinical contexts using non-invasive biofluids. In the following section, we briefly describe selected multiplexed biosensors for the detection of biomarkers relevant to FM that have been evaluated in non-invasive samples such as saliva.

Microelectrode arrays and electrochemical platforms have enabled the simultaneous determination of various biomarkers in sweat and other biofluids, with high sensitivity and minimal crosstalk. Likewise, recent enzymatic biosensors have demonstrated the sensitive detection of catecholamine neurotransmitters in artificial sweat, reinforcing the feasibility of electrochemical monitoring of neurotransmitters in sweat-based matrices [99].

**Figure 4:**
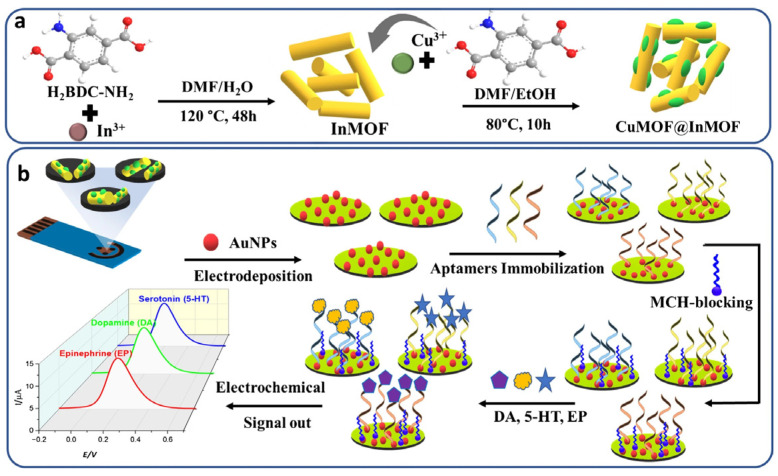
Biosensor Preparation: (**a**) CuMOF@InMOF chemical components and synthesis steps, (**b**) Schematic diagram of the multi-neurotransmitters’ biosensor based on Aptamers-Coupled AuNPs@CuMOF@InMOF Reproduced with permission from [86].

Recently, a wearable aptasensor based on a MOF-on-MOF (CuMOF@InMOF) heterostructure achieved real-time detection of DA, 5-HT, and EN in sweat with low-nanomolar limits of detection (0.18 nM for DA, 0.33 nM for 5-HT, and 0.27 nM for EN) over a broad dynamic range (1 nM–10 µM) [86]. The biosensor employs gold nanoparticle-enhanced electrodes and thiolate nucleic acid aptamers specific to each neurotransmitter, integrated into a flexible, microfluidic patch for continuous sweat collection and analysis. Structural validation confirmed improved surface area, stability, and electron transfer, contributing to high sensitivity and selectivity (Figure 4).

The multiplex cytokine detection has also been reported. In this sense, a multiplexed aptamer-based immunosensor simultaneously measured IL-6 and TNF-α with high sensitivity, in saliva and sweat [87]. The platform was constructed using in-lab printed electrochemical cells, with the working electrode functionalized with gold and platinum nanoparticles to enhance detection performance. Specificity was achieved by immobilizing two distinct aptamers, each labeled with a different redox probe, enabling parallel signal readouts for both biomarkers. The aptasensor exhibited a linear detection range of 5–5000 pg/mL and a limit of detection of 1.6 pg/mL, demonstrating high sensitivity suitable for clinical applications. The dual-target aptasensor was validated using real biological samples, including raw saliva and sweat, collected from both healthy individuals and patients with Long-COVID syndrome, with results successfully cross-verified against ELISA.

**Figure 5:**
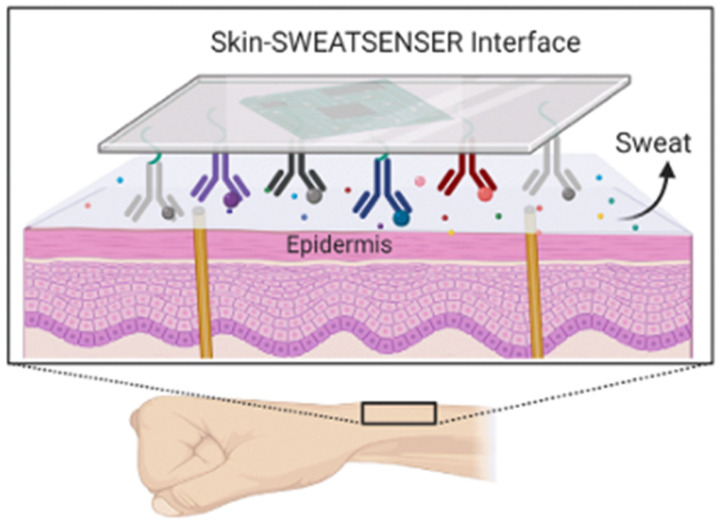
SkinSWEATSENSER device interface. Reproduced with permission from [106].

Another approach involves the simultaneous detection of more than two biomarkers. For example, Jagannath et al. (2021) presented a wearable device capable of real-time, noninvasive monitoring of IL-6, IL-8) IL-10, and TNF-α in passive sweat. The device employs a bioelectronic sensing platform that enables high-sensitivity detection over an analytical range of 0.2–200 pg/mL. Clinical validation demonstrated a strong correlation with standard reference methods, and comparative studies showed consistent IL-8 levels between sweat and serum samples. Importantly, the distinction between healthy and infected individuals (infection or inflammatory symptoms) highlights its potential as a noninvasive tool for personalized monitoring of host immune response, infection progression, and therapeutic efficacy (Figure 5) [106].

Extending integrated multiplexed electrochemical approaches to FM offers a highly promising avenue for simultaneous monitoring of neurochemical, hormonal, metabolic, and inflammatory pathways, enabling the generation of a dynamic, real-time biomarker profile that reflects the syndrome’s complex and heterogeneous pathophysiology. These advancements demonstrate the technical feasibility and translational potential of electrochemical biosensors for minimally invasive monitoring of molecular alterations in FM, spanning neurotransmitters, cytokines, and small-molecule metabolites. Importantly, the versatility of these platforms allows adaptation to emerging FM-associated biomarkers, supporting longitudinal, personalized monitoring and opening new opportunities for mechanistic studies and precision management of this multifaceted chronic condition.

In summary, all biomarkers identified as relevant to FM can, in principle, be monitored using electrochemical platforms. However, several targets—including PGAM1, TALDO1, serotransferrin, calgranulin A (S100A8), serine, and ascorbic acid—currently lack dedicated biosensors, highlighting clear opportunities for technological development. Multiplexed electrochemical biosensing represents the most promising strategy to capture the complex and heterogeneous pathophysiology of FM, enabling simultaneous monitoring of neurochemical, hormonal, metabolic, and inflammatory mediators. Such approaches offer substantial clinical potential for longitudinal patient monitoring, assessment of therapeutic interventions, and mechanistic studies, paving the way toward personalized and precision management of this multifaceted syndrome.

### 3.3. Clinical and Research Value

Building upon the demonstrated technical feasibility for measuring complex biomarker panels, including neuroendocrine regulators (e.g., cortisol), neurotransmitters, metabolites, and protein-level mediators, in accessible biofluids, electrochemical biosensing platforms hold substantial and paradigm-shifting clinical and research potential in the context of FM. The most significant impact lies in enabling high-frequency, temporally resolved longitudinal monitoring using wearable and non-invasive home-based sensors. This capability facilitates the repeated, objective assessment of physiological biomarkers across multi-day or multi-week cycles, allowing for robust cross-correlation analyses with high-resolution symptom fluctuations (e.g., dynamic pain intensity and sleep architecture disturbances). By integrating this dense time-series data, these platforms are poised to move beyond purely symptom-based categorization, enabling the data-driven identification of distinct biological signatures or FM endotypes, such as “inflammatory-dominant” or “HPA axis-sensitive” phenotypes, which are critical for guiding truly personalized and targeted therapeutic strategies and precision drug development. Furthermore, sensors provide the quantitative, objective measure of efficacy required for both pharmacological agents and non-pharmacological interventions, allowing clinicians to swiftly transition from subjective trial-and-error approaches to data-informed therapeutic titration.

An additional aspect that should be considered when translating wearable biosensing technologies to FM monitoring is the physiological characteristics of the patient population. Individuals with FM frequently report heightened cutaneous sensitivity and allodynia, which may affect the long-term tolerability of skin-contact devices, especially those relying on adhesive electrodes or rigid materials. Therefore, the selection of biocompatible, soft, and breathable materials becomes critical to ensure comfort, minimize skin irritation, and promote sustained patient adherence during prolonged monitoring periods. Furthermore, FM has been associated with alterations in autonomic function and sudomotor activity, including abnormal sweat secretion patterns. Such variability may influence the performance of sensors that rely on skin conductance, sweat composition, or stable electrode–skin interfaces, potentially affecting signal stability and analytical accuracy. Consequently, future wearable platforms designed for FM monitoring should incorporate user-centered design strategies, optimized materials, and calibration approaches capable of accounting for inter-individual differences in sweat production and skin sensitivity to ensure reliable long-term physiological monitoring.

From a patient-centered translational perspective, real-time physiological feedback from wearable devices can empower patient self-management, helping individuals to identify and proactively adjust behaviors in response to potential triggers before symptom exacerbation occurs.

Finally, the integration of dense longitudinal datasets, combining biomarker readings, patient-reported outcomes, and environmental or lifestyle information, offers a powerful research tool to advance our fundamental understanding of FM pathophysiology, disease progression heterogeneity, and therapeutic response patterns, addressing the significant diagnostic and management challenges stemming from the current lack of reliable objective markers.

Given the current challenges in FM diagnosis and management, which stem from the subjective nature of symptoms and the lack of reliable objective markers, the adoption of electrochemical biosensors represents a potential paradigm shift toward more precise, data-driven, and truly individualized care in this complex chronic condition.

Nucleic-Acid-Based Electrochemical Biosensing in FM.

Although no genetic or epigenetic biomarker has yet been validated for clinical use in FM, several molecular targets have emerged from exploratory studies. Polymorphisms in neurotransmitter-related genes (e.g., SLC6A4, DRD4) and differential DNA methylation patterns in pain signalling pathways have been reported in FM cohorts. In addition, alterations in specific microRNA expression profiles have been observed in peripheral samples from FM patients. These preliminary findings highlight potential targets for nucleic-acid-based detection strategies, including electrochemical genosensors, and underscore the need for further investigation of genetic and epigenetic biomarkers in multimodal sensing platforms for FM [139,140].

Electrochemical genosensors represent a promising approach for nucleic-acid-based biomarker detection. These devices rely on specific hybridization between immobilized DNA probes and complementary target sequences, translating binding events into measurable electrochemical signals through redox-active reporters.

The integration of nanostructured electrodes, nanostructured catalytic materials, and microfluidic platforms can significantly enhance any sensing system by improving sensitivity, selectivity, and signal amplification. Nanostructured catalytic materials facilitate faster electron transfer and more efficient signal generation, while microfluidics allows precise sample handling and improved mass transport, increasing reproducibility.

Although these strategies have not yet been applied specifically to FM, their success in detecting biomarkers in other clinical contexts demonstrates their feasibility and potential for developing sensors capable of monitoring FM-related biomarkers, even in non-invasive matrices.

In FM research, these platforms could potentially be employed to detect polymorphisms, epigenetic modifications, or microRNA signatures associated with altered pain processing, neurotransmission, or inflammatory regulation. Incorporating these sensors into wearable or minimally invasive devices may enable multimodal, real-time monitoring of molecular changes, ultimately supporting patient stratification and the development of personalized therapeutic strategies.

### 3.4. Technological Frontiers: Novel Materials and Wearable Architectures for FM Monitoring

To translate the clinical potential described above into reliable tools, recent years have seen a paradigm shift in biosensor engineering, moving toward “lab-on-the-skin” platforms that combine high-performance catalysis with mechanical soft-compliance. In the realm of novel catalytic materials, the integration of 2D nanomaterials such as MXenes and transition metal dichalcogenides (TMDs) has significantly surpassed traditional electrodes in terms of effective surface area and electron-transfer kinetics. These materials are particularly relevant for detecting FM-related neurotransmitters and cytokines, which require sub-nanomolar sensitivity in complex non-invasive matrices. Furthermore, the development of metal–organic frameworks (MOFs) and nano-architecture composites allows for the creation of highly selective catalytic sites that can operate in the complex chemical environment of human sweat without rapid degradation [141].

To transition from static to continuous monitoring, the integration of microfluidic systems into wearable formats is now essential. Modern architectures utilize capillary-driven microchannels and “chronological reservoirs” that passively route fresh biofluids—such as sweat or saliva—toward the active sensing area. This ensures that the sensor is constantly exposed to new samples, providing time-stamped physiological data that is crucial for tracking the circadian fluctuations of biomarkers like cortisol. Such microfluidic handling also minimizes evaporative bias and contamination, which have historically plagued on-body electrochemical measurements [142].

Finally, the emergence of flexible and stretchable architecture has addressed the critical issue of patient comfort, especially in individuals with FM-related skin hypersensitivity. Advances in soft bioelectronics, utilizing conductive hydrogels and liquid metal interconnects, allow devices to maintain electrochemical performance even under significant mechanical deformation (stretching, bending, or twisting). These “skin-like” sensors conform to the complex topography of the body, reducing motion artifacts and ensuring stable contact during daily activities or sleep. By combining these stretchable substrates with wireless modules (NFC/Bluetooth), next-generation FM monitors can provide unobtrusive, real-time feedback, bridging the gap between clinical research and the patient’s daily reality.

## 4. Challenges and Limitations

Despite the promising potential of electrochemical biosensors for monitoring FM biomarkers, several hurdles must be cleared before these tools reach routine clinical use. A primary concern is the inherent biological variability of the syndrome. Since FM is highly heterogeneous, patients may present widely different profiles—ranging from inflammatory and neurochemical imbalances to stress-axis dysregulation—making it difficult to establish a “one-size-fits-all” biomarker panel. Furthermore, many relevant analytes circulate at ultra-low concentrations and fluctuate based on circadian rhythms, diet, or sleep patterns. This temporal variability means that single-point measurements are often insufficient; instead, longitudinal designs are required to separate true physiological signals from background biological noise.

From a technical standpoint, the complexity of biofluids like saliva or sweat remains a major barrier. Issues such as electrode biofouling—where the non-specific adsorption of proteins and lipids passivates the surface—can quickly degrade sensor performance. Addressing this requires more than simple surface modification; it demands advanced interface engineering, such as using zwitterionic polymers or poly(ethylene glycol) (PEG) brushes to create a hydration layer that repels foulants while allowing analyte diffusion. Similarly, the presence of electroactive interferents like uric or ascorbic acid in saliva necessitates the integration of permselective membranes (e.g., Nafion^®^ or cellulose acetate) to act as charge-based or physical barriers [143].

Operational stability during continuous monitoring is another critical bottleneck. Signal drift, often caused by the gradual degradation of biorecognition elements or reference electrode instability, can compromise data integrity over time [144]. Potential solutions include adopting differential sensing architectures for real-time background subtraction and stabilizing solid-state reference electrodes with hydrophobic coatings. Beyond general technical hurdles, the physical interface with the FM patient population introduces unique challenges. Patients frequently experience widespread skin hypersensitivity and allodynia; therefore, wearable sensors must move beyond rigid designs toward flexible, breathable, and highly biocompatible materials that ensure patient compliance without triggering discomfort or adverse skin reactions during prolonged wear.

Furthermore, FM is often associated with abnormal sweat secretion rates and electrolyte compositions, which can significantly skew the quantification of target biomarkers. This physiological variability necessitates the implementation of specialized sensor calibration protocols and the integration of multi-modal sensing platforms. Such systems can simultaneously monitor auxiliary parameters—such as local pH, temperature, or skin impedance—to feed multi-modal algorithms that compensate for matrix-induced fluctuations in real-time. By executing this dynamic compensation, the device ensures that the biochemical readout remains accurate and independent of the patient’s individual sudomotor profile or changing environmental conditions.

As discussed in Section 3.4, the high-dimensional and complex data generated by advanced catalytic materials and microfluidic systems require new approaches to data processing. Rather than relying solely on traditional linear analysis, the integration of Artificial Intelligence (AI) and Machine Learning (ML) techniques may be decisive. Algorithms such as Random Forest and Neural Networks are particularly suited to deconvolute overlapping signals, automatically correct sensor drift, and implement multi-modal compensation strategies.

In the context of FM research, these computational approaches could help identify specific “biomarker fingerprints”, correlating biochemical changes with subjective pain scores and enabling personalized flare prediction. By allowing real-time analysis and adaptation to individual variability, AI and ML have the potential to enhance both the reliability and clinical applicability of wearable electrochemical biosensors.

Finally, the lack of published longitudinal studies in FM populations remains a significant gap. Rigorous clinical validation is needed to confirm that biomarker fluctuations captured by sensors reflect disease dynamics. This will require not only technological innovation but also a concerted effort toward standardizing sampling protocols and data normalization. Ultimately, moving from laboratory prototypes to regulated, cost-effective clinical tools will depend on demonstrating a clear benefit in patient management and therapeutic outcomes.

## 5. Conclusions and Future Directions

Fibromyalgia is increasingly recognized not as a single-pathway disease, but as a multifaceted syndrome encompassing heterogeneous biological mechanisms, including central sensitization, neuroendocrine dysregulation, immune-inflammatory activation, metabolic imbalance, oxidative stress, and altered neurotransmission. Consistent with this complexity, a broad spectrum of candidate biomarkers has been reported over the past decades—ranging from neurotransmitters (e.g., 5-HT, NE) and excitatory/inhibitory amino acids (glutamate, GABA), to metabolic and glycolytic enzymes (PGAM1, α-enolase, transaldolase), stress-related proteins (cortisol, α-amylase), oxidative stress markers, iron-binding proteins such as transferrin, and pro-inflammatory cytokines (IL-6, TNF-α).

This molecular diversity reflects the systemic and heterogeneous nature of FM, but it also highlights a translational gap: despite the abundance of proposed biomarkers, their integration into routine clinical practice remains limited. Most have been investigated in blood-derived matrices, whereas exploration in saliva—although highly attractive due to its non-invasive, repeatable, and stress-free collection—remains comparatively constrained. Salivary biomarkers are influenced by circadian rhythms, stress reactivity, flow rate, and lower analyte concentrations, and many lack standardized protocols or validated reference ranges in FM populations.

Given this scenario, longitudinal monitoring of physiological biomarkers—beyond pain scores and self-reported questionnaires—represents a promising strategy to better understand disease dynamics, identify patient subtypes, personalize treatment approaches, and potentially empower patients through objective feedback. Electrochemical biosensors are particularly well suited for this purpose due to their low sample volume requirements, compatibility with minimally invasive or non-invasive matrices, portability, rapid readout, and multiplexing capability. Importantly, several FM-relevant biomarkers—including salivary cortisol, metabolic markers, oxidative stress indicators, and selected proteins—are technically amenable to electrochemical detection, and preliminary platforms such as MIP-based cortisol sensors already demonstrate analytical feasibility.

However, substantial challenges must be addressed before clinical implementation can be realized. These include the development of robust and highly sensitive multiplexed devices, rigorous validation in longitudinal FM cohorts, harmonization of sampling and analytical procedures, and, critically, demonstration of clinical relevance—namely, that biomarker fluctuations correlate meaningfully with symptom severity, flare dynamics, or therapeutic response.

A critical aspect in evaluating the analytical suitability of sensing technologies for FM biomarkers is the relationship between the LOD of available sensors and the actual concentration ranges reported in biological samples. However, a major challenge in this field is the limited availability of quantitative data in non-invasive matrices, such as saliva, sweat, or interstitial fluid, specifically from FM patients. Most concentration values reported for proposed FM-related biomarkers—including IL-6, TNF-α, IL-10, CXCL9/10, cortisol, serotonin, dopamine, and α-amylase—derive from exploratory clinical studies or from measurements performed in serum or plasma, often with substantial inter-individual variability and without universally accepted pathological thresholds. For example, salivary cortisol concentrations typically range between approximately 0.7 and 11 ng/mL, whereas sweat cortisol has been reported in the range of ~8–142 ng/mL, depending on physiological conditions and sampling protocols. In contrast, many current sensing platforms—including immunoassays and other ultrasensitive detection strategies—achieve LOD values in the pg/mL to fg/mL range for cytokines such as IL-6 or TNF-α, and sub-ng/mL levels for cortisol detection, which are well below these general physiological concentrations. From an analytical perspective, this indicates that existing sensing technologies are, in principle, capable of detecting relevant biomarker levels in biological fluids.

Nevertheless, the interpretation of these measurements is further complicated by the high inter-patient variability characteristic of FM, reflecting the heterogeneous and multifactorial nature of the syndrome. In addition, pre-analytical factors related to sample collection and handling can significantly influence the measured concentrations. Variables such as the time of sampling, circadian rhythms (particularly relevant for cortisol), the method used for saliva or sweat collection, and storage or processing conditions may all contribute to variability across studies. This methodological heterogeneity makes direct comparison between datasets difficult and further hinders the establishment of consistent reference ranges.

Consequently, the absence of well-defined concentration ranges in FM patients—particularly in non-invasive matrices—remains a major limitation, as it prevents a definitive assessment of whether current detection limits truly correspond to the pathophysiological variations associated with the disease. Future research should therefore focus not only on improving analytical sensitivity but also on systematically characterizing biomarker concentrations in FM populations and standardizing sampling protocols, which will be essential for translating sensing technologies into clinically meaningful diagnostic and monitoring tools.

Future research should therefore prioritize the validation of these biosensing platforms in well-characterized FM patient cohorts, together with standardized sampling protocols and longitudinal monitoring strategies. Such efforts will be essential to bridge the gap between promising laboratory demonstrations and clinically implementable biosensor systems for personalized disease monitoring.

Ultimately, integrating multi-biomarker electrochemical platforms into FM care aligns with the broader shift toward personalized, data-driven, patient-centered management of chronic complex disorders. Moving beyond purely subjective symptom tracking toward objective physiological monitoring could redefine how disease activity is assessed and managed in FM.

## Data Availability

No new data were created or analyzed in this study. Data sharing is not applicable to this article.
